# The Orexin System in Addiction: Neuromodulatory Interactions and Therapeutic Potential

**DOI:** 10.3390/brainsci15101105

**Published:** 2025-10-14

**Authors:** Toni Capó, Jaume Lillo, Joan Biel Rebassa, Pau Badia, Iu Raïch, Erik Cubeles-Juberias, Irene Reyes-Resina, Gemma Navarro

**Affiliations:** 1 Department of Biochemistry and Physiology, School of Pharmacy and Food Sciences, University of Barcelona, 08028 Barcelona, Spain; tonicapoquetglas@ub.edu (T.C.); jaumelillo@ub.edu (J.L.); jrebaspa7@alumnes.ub.edu (J.B.R.); pbadiafe47@alumnes.ub.edu (P.B.); iuraichipanisello@gmail.com (I.R.); ecubelju7@alumnes.ub.edu (E.C.-J.); ireyesre8@ub.edu (I.R.-R.); 2 Network Center for Biomedical Research in Neurodegenerative Diseases, CiberNed, Spanish National Health Institute Carlos III, Av. Monforte de Lemos, 3–5, 28029 Madrid, Spain; 3 Institut de Neurociències UB, Campus Mundet, Passeig de la Vall d’Hebron 171, 08035 Barcelona, Spain

**Keywords:** drug addiction, orexin A, orexin B, orexin receptor 1 (OX_1_R), orexin receptor 2 (OX_2_R)

## Abstract

According to the World Drug Report, there are nearly 300 million drug users globally. Drug addiction is a chronic, relapsing brain disease that leads to medical, psychological, and social complications. This neuropsychiatric disorder is characterized by a compulsive drug-seeking behavior, continued use despite harmful consequence, and long-lasting changes in the brain. The reward system, which involves dopaminergic circuits, plays a key role in addiction. Dopamine levels have been described to fluctuate throughout the day, in a circadian fashion, and the effects of drugs have been shown to depend on the time when they are used. Hence, due to its important role in the control of circadian rhythms, the orexinergic system seems to have a role in the regulation of addiction. This system is composed by the orexin receptors 1 and 2 (OX_1_R and OX_2_R), the ligands orexin A (OXA) and orexin B (OXB) and their respective enzymes for degradation or synthesis. Here, we explore how orexin receptors and orexin peptides are involved in addiction. For instance, OX_1_R has been shown to be strongly involved in specific behaviors such as drug-seeking for stimulants, alcohol and other addiction problems, whereas OX_2_R appears to be linked with arousal and stress responses. We also investigate how the orexinergic system may regulate drug-seeking behavior by interaction with other brain systems such as the dopaminergic, cannabinoid or opioid systems. Finally, the potential of receptor complexes as new therapeutic targets to treat drug addiction is explored.

## 1. Introduction

Drug addiction constitutes one of the most complex and persistent public health problems, affecting not only those who suffer from it but also their families and communities. Unfortunately, drug addiction remains a global issue today. Studies estimate that in 2023, around 316 million people (about 6% of the global population between 15 and 65 years old) consumed some type of drug, excluding alcohol and tobacco. Trends indicate that global drug use has worsened since 2013, as incidence has risen from 5.2% to today’s 6% [1]. Addiction is considered a chronic brain disorder, and it involves rewiring of brain circuitry and therefore a change in its functionality [2]. This pathology is characterized by a drug-seeking behavior, an impairment of self-control and the emergence of a negative emotional state when drug access is limited [3,4]. Addiction to a substance relies on several mechanisms, and the potency of the addiction mostly depends on the drug abused, individuals’ genetics and environmental cues, which play a significant role in the addictive process [5,6].

To understand how addiction works at a molecular level, it is important to comprehend the mechanisms that drive animal motivation and enhance animals’ desire to obtain certain stimuli. The reward system (also known as the mesolimbic system) is a primary mechanism present in all animals as a way to survive, as it is capable of motivating the animal to obtain food, reproduce, drink, etc. [7,8]. It consists of dopaminergic neuron projections that originate at the ventral tegmental area (VTA) and extend to the striatum, prefrontal cortex, amygdala, hippocampus, and additional regions of the limbic system [8]. The main physiological function of the reward system is to associate a stimulus with a positive outcome [7]. The first encounter with the stimulus establishes the association in the reward system thanks to the plasticity provided by dopaminergic activity, rewiring Central Nervous System (CNS) structure based on the stimuli. Further encounters with the stimulus will enhance the strength of the association. Privation of stimulus causes the reward system to increase the motivation or drive of the animal to obtain said stimulus [9,10].

The reward system relies on the dopaminergic system in order to work. This system involves the dopaminergic receptors (D_1_, D_2_, D_3_, D_4_ and D_5_ receptors), the neurotransmitter dopamine and the enzymes involved in their degradation and synthesis. Dopamine is the main responsible for learning and reinforcement of these associations made between stimuli and positive outcomes [8], as increases in the levels of dopamine drive the increase in motivation and arousal. When the release of dopamine in the CNS is abnormally high, addiction appears [11]. This increase in dopamine levels is attained via a higher release of neurotransmitter vesicles, a blockade of the reuptake mechanisms and/or an enhanced firing of dopaminergic neurons. The main brain area affected is the striatum, innervated by the reward system, where the dopaminergic receptors 1 and 2 (D_1_R and D_2_R) are most present [5,12,13]. This sudden increase in dopamine after drug use is able to rewire brain connectivity and strengthens the association between the environmental cues and the pleasant feelings when taking the drug, establishing/reinforcing the reward obtained by these substances [2,12].

The orexigenic system (also known as the hypocretin system) is the main responsible for circadian rhythm regulation, but it is also involved in other functions such as feeding, thermoregulation and cardiovascular and neuroendocrine regulation, etc. It is formed by the orexin OX_1_ and OX_2_ receptors, the ligands orexin A and orexin B and their respective enzymes for degradation or synthesis [14]. Orexins were first shown to be implicated in narcolepsy, insomnia [15,16,17,18,19,20,21] and stress responses [22,23,24,25], but it is worth mentioning that orexins have also been described to play major roles in other CNS disorders, comprising both neuropsychologic and neurodegenerative diseases. Thus, orexin system dysregulation has been associated with anxiety [26,27,28,29,30], fear [31,32,33,34,35], depression [36,37,38,39,40,41,42], schizophrenia [43,44,45,46,47], ischemic stroke [48,49,50,51,52], Alzheimer’s disease [53,54,55,56,57,58,59], Parkinson’s disease [60,61,62,63,64] and Huntington’s disease [65,66,67,68]. However, exploring this topic in depth is out of the scope of this work. Details of the involvement of the orexigenic system in these disorders can be found in reviews by Ten-Blanco and collaborators [69] or by Wang et al. [70]. However, it has also been hypothesized that orexins may be important in addiction, as it has been found that dopamine levels change throughout the day, in a circadian fashion, and that the effects of drugs depend on the time when they are used [8,71,72]. There is increasing evidence that indicates that the orexin system plays an important role in addiction to substances such as opioids, cocaine, cannabinoids or alcohol (see the table in Section 3). For example, Alcohol Use Disorder (AUD) produces alterations in orexin expression and receptor activity [73,74], suggesting that this system modulates alcohol intake and participates in the reward and stress related circuits. Therefore, in this review we aim to elucidate how the orexigenic system may be related to drug abuse and addiction, and how it could regulate drug-seeking behavior and relapse by interaction with another signaling systems.

## 2. Orexin Receptors

**Figure 1:**
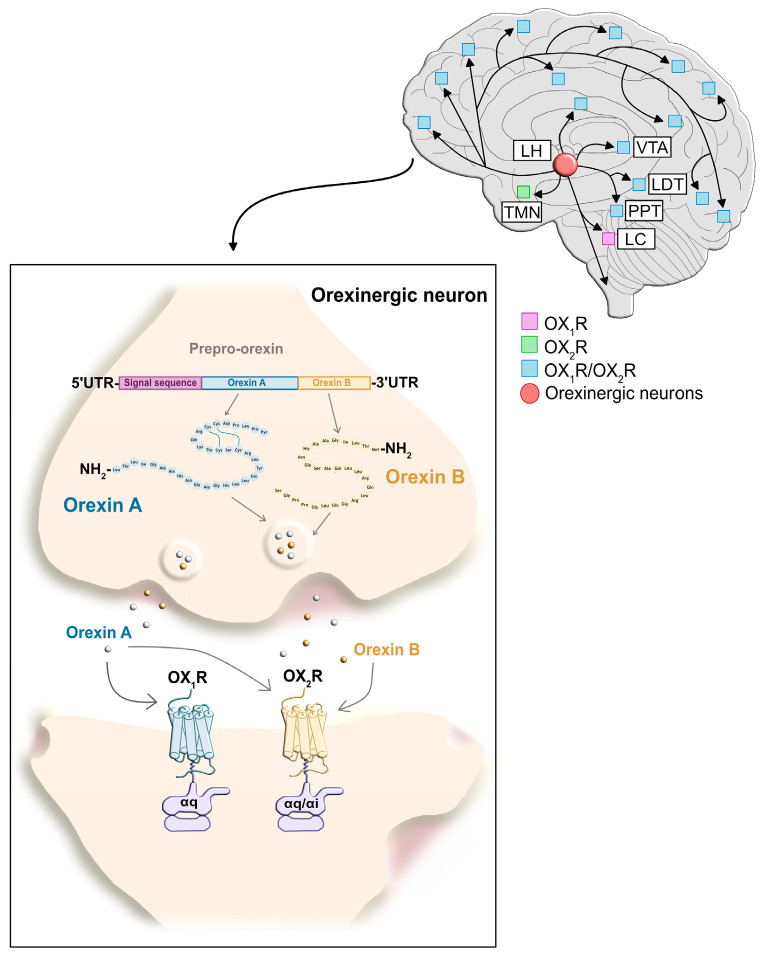
Distribution of OXR expression across different brain regions and representation of the mechanism of action of orexins at the neuronal synapse.

Orexin A and orexin B act on the OX_1_ and OX_2_ receptors. Both receptors belong to the class A subfamily of G protein-coupled receptors (GPCRs) and are composed of 425 and 444 amino acids, respectively, with a high degree of conservation among mammals [75]. The first X-ray crystal structure of OXR was obtained with the antagonist Suvorexant bound, and revealed that the main structural difference between OX_1_R and OX_2_R lies in the presence of an α-helix in the N-terminal extracellular domain of OX_1_R, which may be critical for interaction with the two native peptides [76]. Nevertheless, the two receptors share 64% amino acid sequence homology. At the expression level, OX_1_R has been detected in the kidney, adrenal and thyroid glands, testes, ovaries, and jejunum, whereas OX_2_R is expressed in the lung, adrenal glands, and pituitary [77]. However, most studies focus on the brain, where both receptors are co-expressed in different regions of the CNS, including the VTA, the raphe nuclei, the amygdala, the cortex, and the pedunculopontine (PPT) and laterodorsal (LDT) tegmental nuclei [70]. While OX_1_R expression is restricted to the locus coeruleus (LC), OX_2_R is exclusively expressed in the tuberomammillary nucleus (TMN) (Figure 1) [78].

Orexins are produced by a population of neurons located in the lateral and posterior hypothalamic areas of the human brain [79]. These orexinergic neurons project onto monoaminergic neurons situated in the limbic system, the cerebral cortex, and the brainstem, which are involved in the regulation of the sleep–wake cycle, the maintenance of homeostasis, and the reward system [77]. In turn, orexinergic neurons respond to a wide variety of neuronal signals, as they receive projections from the amygdala and cortex in response to stress, from the nucleus accumbens (NAc) and the VTA to regulate reward and motivation, and from the ventrolateral preoptic nucleus of the hypothalamus to regulate the sleep–wake cycle and circadian rhythms [78].

### 2.1. The Orexin Receptor Type 1

The OX_1_R is encoded by the *HCRTR1* gene, located on chromosome 1, and in humans it is mainly expressed in the hypothalamus, locus coeruleus, and amygdala. OX_1_R plays a key role in the regulation of feeding behavior, wakefulness, motivation, and reward. Activation of OX_1_R in locus coeruleus neurons increases neuronal excitability, thereby facilitating wakefulness and attention. In the hypothalamus, OX_1_R expression is associated with food intake, contributing to the sensation of hunger and the motivation to obtain food. Finally, OX_1_R activation in the VTA enhances dopaminergic transmission, facilitates reward-associated learning, and is implicated in drug abuse sensitization.

OX_1_R mediates the effects of its endogenous ligands, showing higher affinity for OXA (IC_50_ 20 nM) than for OXB (IC_50_ 420 nM) [78]. Although OXRs are generally distributed across different brain regions, OX_1_R predominates in areas involved in appetite regulation [69]. Selective OX_1_R antagonists have been investigated in vivo for the treatment of obesity, anxiety, and addiction [80]. In fact, administration of the selective OX_1_R antagonist SB-334867 reduces food intake and promotes an obese phenotype in leptin-deficient mice [81]. It has been observed that during fasting, prepro-orexin mRNA levels double and orexinergic neurons increase their activation [82]. Transgenic mice lacking orexin neurons do not respond to fasting, indicating that hunger induction requires the orexinergic system [83]. Indeed, orexinergic neurons respond directly to metabolic signals, as they are activated by ghrelin or low glucose levels and inhibited by leptin [84].

### 2.2. The Orexin Receptor Type 2

OX_2_R is expressed in all vertebrate organisms, in contrast to OX_1_R, which is found exclusively in mammals. This suggests that OX_1_R may have emerged as a product of biological evolution [85]. In humans, the gene encoding OX_2_R is *HCRTR2*, located on chromosome 6. OX_2_R is mainly expressed in the CNS, particularly in regions involved in wakefulness, arousal, and metabolic regulation, such as the tuberomammillary nucleus, raphe nuclei, and locus coeruleus [78]. OX_2_R is considered a non-selective receptor, as it shows high affinity for both OXA (IC_50_ 38 nM) and OXB (IC_50_ 36 nM). Upon activation, it couples to the Gαq/11 protein, activating PLC and generating IP_3_ and DAG, which increase intracellular Ca^2+^ levels and thereby modulate neuronal excitability. In addition, OX_2_R can activate the MAPK pathway, promoting neuronal proliferation, survival, and plasticity [86]. Willie, J.T. et al. demonstrated that OX_2_R knockout (KO) mice developed narcolepsy, whereas OX_1_R KO mice exhibited only mild fragmentation of the sleep–wake cycle, indicating that OX_1_R plays a minor role compared to OX_2_R in sleep–wake regulation [87]. Interestingly, OX_2_R KO mice displayed cataplexy and alterations in NREM sleep, while OX_1_R KO mice presented effects on REM sleep [88]. Moreover, intravenous administration of OXA and OXB has been shown to increase wakefulness and reduce sleep duration [89].

### 2.3. Orexin Receptor Signaling: Regulation of Neuronal Excitability and Synaptic Plasticity

As mentioned above, downstream signaling from orexin receptors is diverse. Key pathways include G-protein coupled pathways and the regulation of ion channels that lead to the modulation of neuronal excitability and synaptic plasticity, thereby enhancing addictive memory.

OXRs have been described to couple to G_q_, G_i/o_ and G_s_ and proteins in rat brain (see [90] for details). The Gαq/11 pathway activates phospholipase C (PLC), leading to the production of inositol trisphosphate and diacylglycerol, which increase intracellular calcium (iCa^2+^) levels and activate protein kinase C (PKC). Orexin, via OXRs, has been shown to elevate iCa^2+^ and induce membrane depolarization through the PLC–PKC pathway [90,91]. Ca^2+^ is critical for regulating neuronal excitability by altering ion channel activity and influencing the trafficking of receptors like AMPA receptors to the postsynaptic membrane. Regarding Gαs and Gαi/o pathways, OA and OB stimulation caused OX_2_R to couple to G_i_ proteins in rat cortical neurons, leading to inhibition of cAMP formation [92], while OA caused OX_1_R to stimulate cAMP synthesis in cultured rat astrocytes [93]. Beyond PLC, orexin receptors activate phospholipase A2 [94] and phospholipase D [95] after Gq or Gi protein activation, ultimately resulting in an increase in iCa^2+^ and a downstream cascade response.

OXR activation can also regulate intracellular ion concentration through different ion channels in the CNS, contributing to neuronal depolarization and excitability. Orexin signaling activates non-selective cation channels, as OXA was shown to bind OX_1_R and elevate iCa^2+^ by activating the transient receptor potential channel 3 [96]. Also, orexin signaling inhibits K^+^ channels, as OXA depolarized membrane potential and increased the firing activity of cultured LC and TMN neurons through suppression of G protein-coupled inwardly rectifying K^+^ channels [97]. In addition, orexin has been reported to induce depolarization in TMN neurons through the activation of electrogenic Na^+^/Ca^2+^ exchangers in histaminergic neurons of the TMN [98]. Moreover, the orexin-mediated Ca^2+^ elevation in other neuronal types is mediated by depolarization and the activation of voltage-gated Ca^2+^ channels (VGCCs). Via OX_1_R, OA elevates iCa^2+^ by activating L- and N-type Ca^2+^ channels in dopaminergic [99], prefrontal cortex (PFC) [100] or hypothalamic [101] neurons through the PLC–PKC signaling pathway. Influx of Ca^2+^ through VGCCs activates signaling cascades, such as those involving CaMKII, which can affect receptor exocytosis.

Orexin signaling can also activate protein kinases, including mitogen-activated protein kinases (MAPKs) like ERKs and p38, and the Mammalian Target of Rapamycin complex 1 (mTORC1). Orexins activate the p38-MAPK signaling pathway and increase the level of phosphorylated ERK1/2 [102,103,104]. ERK_1/2_ activation induced by orexins involves Gq/PLC/PKC signaling, but not the protein kinase A pathway [105]. The MAPK pathway is known for regulating various cellular processes, including those involved in synaptic plasticity. In addition, orexins stimulated Akt kinase activation in rat cortical neurons [106]. Orexin signaling has been shown to rapidly activates the mTORC1 pathway, which is dependent on transient cytoplasmic Ca^2+^ [107].

Orexin receptors also regulate synaptic plasticity, impacting on addictive memory. Remarkably, in the VTA, this plasticity is critical to behavioral sensitization resulting from cocaine administration [108] and involves an initial but transient increase in the number of postsynaptic N-methyl-D-aspartate (NMDA)-type glutamate receptors. It has been shown that orexins potentiate the NMDA receptor-driven release of noradrenaline from LC neurons [109]. In the VTA, a similar potentiation of NMDA receptor-mediated synaptic currents was produced by orexin-B [110]. Data indicate that this the enhancement of NMDA receptor current is mediated by the PLC–PKC pathway [108,109].

In summary, endogenous orexin receptors across the CNS promote membrane depolarization and elevate iCa^2+^. Findings further suggest that these receptors play a significant role in modulating cellular excitability through various mechanisms and in regulating synaptic plasticity.

## 3. Different Roles of OX_1_ and OX_2_ Receptors in Addiction: Insights in Orexin Receptor Antagonists

The abuse of addictive substances such as alcohol, cocaine, or opioids has been shown to induce alterations in the orexinergic system. These dysfunctional changes are associated with compulsive drug intake and increased vulnerability to relapse. While OX_1_R is primarily involved in reward-seeking behaviors, OX_2_R is more closely linked to arousal and regulation of the sleep–wake cycle [111]. Consistent with this, individuals suffering substance use disorders frequently exhibit sleep impairments or insomnia, supporting the involvement of the orexin system in the pathophysiology of addiction [112]. Furthermore, the lateral hypothalamus (LH), where OX_1_R is expressed, has been closely associated with the reward system [70], and the orexinergic system—particularly through OX_1_R—has been described to be strongly linked to addiction to substances of abuse such as alcohol [113], nicotine [114] and cocaine [115].

Given the link between drug addiction and increased activity of the orexin system, orexin receptor antagonists have emerged as a promising therapeutic approach for substance use disorders. Growing evidence indicates that blocking either OX_1_R or OX_2_R may help to reduce drug consumption and prevent relapse as well as tolerance and dependence caused by drug abuse [116]. A clear example is provided by studies using the OX_1_R antagonist SB-334867, which, when administered intraperitoneally, attenuated nicotine withdrawal [117], whereas the OX_2_R antagonist TCS-OX2-29 did not. Pretreatment with SB-334867 also reduces amphetamine-induced dopamine release in the NAc [118]. In the study by Hutcheson et al., treatment with SB-334867 blocked d-amphetamine-induced conditioned place preference and decreased responses to cocaine-paired cues in rats [119]. These findings suggest that OX_1_R antagonism could be a promising therapeutic strategy to reduce the impact of environmental cues that drive compulsive drug-seeking behavior in humans.

Cocaine consumption produces an increase in orexin-producing neurons in hypothalamus, and chronic cocaine use also increases the number of orexin receptor-expressing cells. Specifically, in the posterior paraventricular nucleus of the thalamus (pPVT), OX_2_R expression increased in rats after two weeks of cocaine abstinence. Interestingly, OX_2_R expression returned to basal levels after one month of withdrawal [120]. Regarding OX_1_R, knockdown of this receptor resulted in a reduced dopaminergic response to cocaine, as well as a decreased motivation to seek the drug in a mice model [121]. OX_1_R signaling has been shown to mediate multiple cocaine-associated behaviors such as cue-induced reinstatement of extinguished cocaine seeking and the expression of conditioned place preference [122]. Interestingly, pharmacological blockade of OX_1_R with the selective antagonist SB-334867 reduced motivation for cocaine, particularly in animals with high baseline motivation for drug [123].

However, cocaine is not the only drug capable of dysregulating the orexin system. Blood samples obtained from human patients with alcohol dependence showed increased levels of orexin in early stages after withdrawal [124]. Similarly, in a rodent model of alcohol dependence induced by intermittent alcohol vapor exposure, an increase in orexin mRNA expression was observed in the hypothalamus [125]. In line with these results, adult rats that presented acute excessive patterns of alcohol intoxication during adolescence exhibited an upregulation of the orexin system [126]. Moreover, rats that exhibited high novelty-induced locomotor activity, a behavioral trait linked to high alcohol consumption, showed a similar enhancement of orexin signaling [127].

The impact of chronic alcohol use over orexin receptors mRNA expression levels has been addressed in several studies. In a rodent model of alcohol dependence induced by intermittent alcohol vapor exposure both OX_1_R and OX_2_R mRNA expression levels were increased in the pPVT and detectable as early as 8 h after withdrawal [73]. Additionally, pre-fertilization maternal alcohol consumption in zebrafish significantly increased the number of orexin-expressing neurons and alcohol consumption in offspring [128]. Although most studies report an increased expression of orexin receptors, others have observed either reductions or no significant changes [125,129,130,131]. For example, Alcaraz-Iborra and colleagues detected different patterns of OX_1_R expression in C57BL/6J mice. Alcohol consumption induced an increased expression of OX_1_R in the medial prefrontal cortex but decreased expression in the nucleus accumbens [74].

Several studies have investigated the use of orexin receptors antagonists to ameliorate AUD pathology. For example, treatment with JNJ-10397049, a selective OX_2_R antagonist, reduced ethanol self-administration without altering dopamine levels or showing withdrawal signs in rats [132]. Similar results were obtained with a selective OX_1_R antagonist, where SB-334867 significantly reduced ethanol intake and blood ethanol levels in animal models compared to vehicle-injected controls [133], and dual orexin receptor antagonists also showed strong effects [134].

Regarding clinical studies, Campbell et al., reported a case of successful AUD treatment using suvorexant in a patient with comorbid insomnia. Treatment with suvorexant improved sleep quality, craving reduction, and overall functioning, achieving a sustained abstinence during all follow-up visits [135].

Orexin neurons are found in key reward processing regions, such as the VTA and the NAc [136]. In these regions, orexin activation produces dopamine release and enhances reward-seeking cues [137]. Besides its role in reward, the orexigenic system plays an important role also in stress-induced relapse. Acute stressors can activate orexigenic neurons in the hypothalamus, which once activated can trigger the reinstatement of alcohol-seeking behavior even after prolonged abstinence [138]. Interestingly, this stress induced pathway is especially relevant in individuals with a history of alcohol dependence, where blocking orexin receptors has been shown to prevent stress-induced relapse [139].

Opioids are a type of medication used in the management of pain thanks to its potent analgesic effects. Although opioids were originally produced for medical purposes, the euphoric and well-being effects associated with opioid consumption led to abusive non-medical use creating an opioid crisis in different countries worldwide, with devastating consequences for public health. The orexin system is reported to also be affected by opioids. In postmortem brains from heroin addicts, an increased number of orexin-producing neurons compared to non-addict subjects was reported [140]. Interestingly, both mRNA levels of μ-opioid receptor (μOR) and orexin were enhanced by morphine withdrawal [141].

Initially developed for the treatment of insomnia, dual orexin receptor antagonists (DORAs) such as suvorexant, lemboraxant or almorexant are also being investigated for their potential role in treating substance use disorders. Evidence suggests that the administration of almorexant attenuates cocaine-induced inhibition of dopamine uptake and reduces cocaine self-administration [142]. Moreover, suvorexant administered systemically or directly into VTA attenuates the motivational and hedonic properties of cocaine and impulsive cocaine-seeking [143].

Regarding clinical trials using DORAs, a randomized, double-blind, placebo-controlled trial aimed to evaluate the use of suvorexant as a safe and effective pharmacotherapy to treat sleep disorders in alcohol dependent patients undergoing acute alcohol withdrawal and thereafter for six months. The study also aimed to examine the effectiveness of suvorexant in reducing craving for alcohol and promoting duration of abstinence. However, due to supplying problems the study was terminated and no results have been published [144]. Another trial aimed to evaluate whether suvorexant could improve sleep and promote abstinence in individuals with opioid use disorder. Although it was in phase 2, it was terminated prematurely due to feasibility and enrollment issues, and no outcome data have been released [145]. Lemborexant has also been explored in a phase 1b/2a safety study as an adjunctive treatment for insomnia to buprenorphine-naloxone for opioid use disorder. Results support the tolerability of lemborexant as an adjunctive treatment for insomnia in humans [146]. A human abuse potential study, daridorexant showed dose-related drug-liking among recreational sedative drug users with lower effects at the highest phase-3 dose, and similar effects at higher doses compared to supratherapeutic doses of suvorexant and zolpidem [147]. As DORAs affect brain systems involved in reward, their abuse potential needs careful monitoring, although initial assessments generally indicate a low abuse liability compared to traditional hypnotics. However, most of the currently available data have been derived from preclinical studies, and future work is needed to evaluate the efficacy of orexin receptor antagonists for the clinical treatment of addiction [148].

Besides addiction, DORAs are also being investigated for their broader therapeutic potential in various neuropsychiatric disorders, including depression, bipolar disorder, migraine and substance use disorders [149]. By improving sleep quality, DORAs can indirectly alleviate symptoms associated with these comorbid conditions. Clinical research conducted on patients with insomnia, anxiety, and depression, suvorexant improved not only insomnia but also anxiety symptoms [150]. Moreover, pre-clinical studies have shown that DORAs can attenuate post-traumatic stress disorder [151,152]. Additionally, suvorexant in treatment with the antipsychotic aripiprazole may be useful in treating insomnia comorbid with schizophrenia, suggesting a broader application in severe mental illnesses [153]. In addition, potent selective brain-penetrant OX_1_R antagonists with anxiolytic effects have been discovered, such as ACT-335827, which shows high selectivity for OX_1_R across different neuronal cell types and has a pharmacokinetic profile suitable for oral in vivo administration. ACT-335827 produces mild anxiolytic effects in rats without affecting motor or cognitive function and without reducing wakefulness [154]. Therefore, ACT-335827 may serve as a novel pharmacological tool to further explore the specific role of OX_1_R signaling in physiology, behavior, and addiction.

Hence, the use of orexin receptor antagonists appears as a promising approach for therapeutic intervention. Table 1 summarizes relevant preclinical studies in which the orexin system has been targeted to investigate the addiction to different substances of abuse. Future research should focus on receptor-specific functions across different substances, and explore the role of OX_1_R signaling, alone and combined with OX_2_R signaling, to better understand the of the orexin system in substance abuse disorders.

## 4. Implication of Orexin Peptides in Addiction

As indicated above, there is cumulative evidence showing that drug exposure increases orexin production. A persistent upregulation of orexin levels in LH neurons has been detected in animal models of drug addiction and suggested in humans. Interestingly, when the levels of orexin were normalized, a reverse in drug motivation was observed [110,182,192]. This maladaptive activation of orexinergic pathways increases drug salience and promotes compulsive drug-seeking behavior. Moreover, repeated drug exposure has been shown to induce a long-lasting, experience-dependent potentiation of glutamatergic synapses on orexin-producing neurons in mice [193].

**Figure 2:**
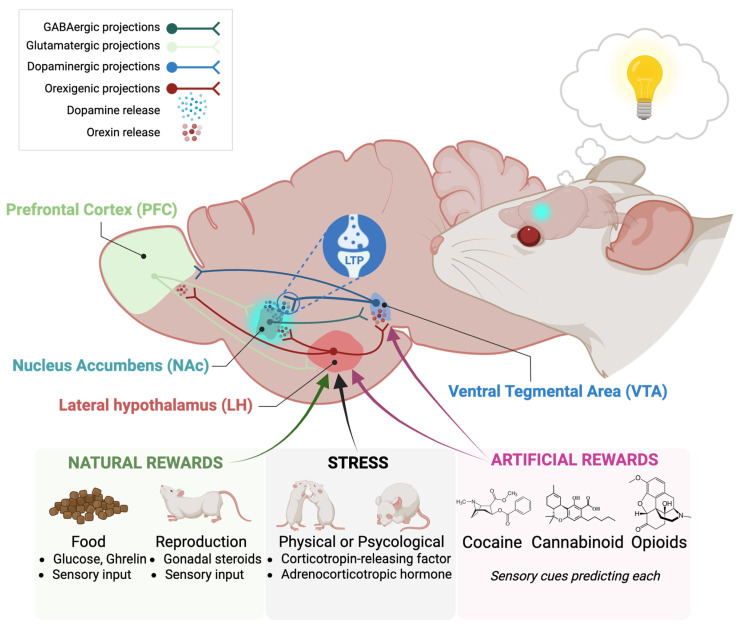
Schematic representation of the orexigenic system as a regulator of the mesolimbic reward circuit. The lateral hypothalamus sends orexinergic projections to the ventral tegmental area, the nucleus accumbens and the prefrontal cortex, modulating dopaminergic signaling and synaptic plasticity within the pathway. In parallel, the PFC exerts top-down control through glutamatergic projections to the NAc, which also sends GABAergic projections to the VTA, shaping reward-related decision-making. Natural rewards (food, reproduction), artificial rewards (cocaine, cannabinoids, opioids), and stress (physical or psychological) act as inputs to this system, altering the activity of both orexinergic neurons in the LH and dopaminergic neurons in the VTA. Importantly, orexin-driven activation of VTA dopaminergic neurons promotes long-term potentiation (LTP) between VTA and NAc neurons, reinforcing the reward circuit’s activation pattern. This plasticity generates a predisposition toward reward-seeking behaviors, which can ultimately drive the transition from physiological reward processing to pathological addiction.

Over the past decades, it has been established that the orexin system acts as an active modulator of the neurosignaling and neurotransmission in addictions [194]. It has been demonstrated that orexins have a crucial role in the neuromodulatory interaction with glutamate, dopamine, GABA, and endocannabinoid systems [195,196]. For instance, it was reported that orexins released during stress, via OX_1_R, contributed to the reinstatement of cocaine seeking through endocannabinoid/CB_1_ receptor (CB_1_R)-mediated dopaminergic disinhibition in the VTA in mice. Specifically, restraint stress activated hypothalamic orexin neurons, which release orexins into the VTA to activate postsynaptic OX_1_Rs of dopaminergic neurons. The activation of OX_1_Rs resulted in 2-arachidonoylglycerol (2-AG) synthesis through a G_q_-protein signaling cascade. 2-AG retrogradely inhibited GABA release through presynaptic CB_1_Rs, leading to VTA dopaminergic disinhibition and reinstatement of cocaine seeking [183,197]. These data suggest that orexin regulates other neurotransmitter systems such as the dopaminergic system in addiction environments (Figure 2).

New findings further support that orexin modulates excitatory synaptic transmission and plasticity, thus altering glutamatergic/GABAergic balance. This provides a mechanistic link between orexin and drug-induced plasticity [198]. In a randomized trial, the administration of a dual orexin receptor antagonist among individuals undergoing opioid withdrawal lowered diurnal salivary cortisol levels compared to placebo, and reduced self-reported stress, supporting the relevance of the orexin’s role in modulating stress and other mechanisms related to addiction and abstinence [163,199]. Hence, clinical findings in human studies highlight the necessity of targeting the orexin system for the treatment of addiction.

However, there are important limitations and controversies that must be acknowledged. Even though the initial human laboratory and pilot studies are being truly promising, on the other side, the clinical evidence remains a step behind, there is still a limitation on controlled trials and its efficacy of evaluating orexin antagonists used in disorders.

## 5. Orexin System Contribution to Relapse of Natural Rewards and Stress-Reward Circuitry

Orexins are intrinsically involved in natural rewards like feeding and sexual behavior. Initially recognized for stimulating food intake, orexin neurons activate robustly in response to palatable food and its anticipation, driving food-seeking motivation [200,201]. Orexin signaling mediates reward-based feeding and can regulate cue-induced overconsumption [202]. Similarly, orexin neuron activity increases during male copulation [203]. While not directly impacting sexual performance, orexin cell lesions alter conditioned responses to sexual reward, suggesting a role in associating environmental cues with pleasurable experiences [200].

The orexin system also plays a pivotal role in integrating arousal, motivation, and reward processes, with its widespread projections to key brain regions involved in the stress and reward circuitry, such as the VTA, NAc, PFC, LC, and amygdala, positioning it as a critical modulator of drug-seeking behavior and relapse [204] (Figure 2). This system is intimately linked with the stress response, particularly through its interactions with the corticotropin-releasing factor (CRF) system and the hypothalamic–pituitary–adrenal (HPA) axis, as stress is a major precipitant of drug relapse and orexin neurons are highly responsive to various stressors [205]. Orexin neurons receive direct input from CRF-containing neurons and express CRF receptors, meaning that activation of the CRF system can directly activate orexin neurons, leading to increased orexin release and suggesting that stress-induced activation of CRF can recruit the orexin system to promote arousal and drug-seeking behaviors [206]. Furthermore, orexin neurons project to hypothalamic nuclei that regulate the HPA axis, and orexin administration can stimulate HPA axis activity, establishing a reciprocal relationship where stress-induced HPA axis activation can influence orexin signaling, and conversely, orexin can impact the physiological stress response, an interplay crucial in the context of addiction where chronic stress and HPA axis dysregulation are common [206].

The orexin system’s involvement in the stress-reward circuitry makes it a significant contributor to drug relapse, which is often triggered by stress, drug-associated cues, or re-exposure to the drug itself, as orexins enhance the motivational salience of rewards, including drugs of abuse, and promote arousal and attention towards drug-related stimuli [207]. Experimental evidence strongly supports a role for orexins in stress-induced relapse, with studies showing that acute restraint stress activates lateral hypothalamic orexin neurons, and blockade of orexin receptors can attenuate stress-induced reinstatement of cocaine-seeking behavior in rodents, suggesting that stress activates the orexin system, which in turn drives the motivation to seek drugs as a coping mechanism [183,208]. Orexin neurons are also activated by drug-associated cues and contexts, and their projections to reward-related areas like the VTA and NAc are critical for mediating the motivational effects of these cues, thereby contributing to cue-induced relapse by potentiating dopamine release in the NAc, a key mechanism underlying the reinforcing effects of drugs and the motivational drive for drug seeking [209,210]. Re-exposure to drugs of abuse can also activate the orexin system, further promoting drug-seeking, suggesting a feed-forward loop where drug exposure activates orexin, which then enhances the rewarding properties of the drug and the motivation to consume more [192].

The intricate interplay between the orexin system, the CRF system, and the HPA axis thus provides a robust neurobiological context for understanding the vulnerability to relapse in addiction, as by modulating arousal, attention, and the emotional response to stress, orexins can tip the balance towards drug-seeking behaviors, especially under challenging conditions, with targeting the orexin system, particularly OX_1_R, showing promise in preclinical models for reducing drug intake and preventing relapse across various substances of abuse, highlighting its potential as a therapeutic target for addiction treatment [211,212].

## 6. Orexin System Interactions with Other Systems in Addiction

The orexigenic system, a key neuromodulatory system, exerts significant influence on other neurotransmitter systems [204,213]. This regulatory capacity makes it a critical player in complex brain disorders like addiction. The orexin system is particularly engaged by stimuli associated with rewards, including abuse drugs, and interference with OX_1_R neurotransmission can block drug-seeking behaviors [198]. This section will delve into the intricate interactions between the orexigenic system and other important GPCR systems, including opioid, dopaminergic, and cannabinoid systems, highlighting their synergistic and sometimes antagonistic roles in the context of addiction.

### 6.1. Orexin-Opioid System Interaction

The interplay between the orexigenic and opioid systems is particularly relevant in the context of opioid addiction [158,162]. Opioid use disorders are characterized by intense craving and relapse [214], and the orexin system has been shown to play a significant role in mediating these behaviors [162,215]. The orexin system’s influence on the mesolimbic dopamine system, a key pathway in reward and addiction, is a major mechanism through which orexin modulates opioid-related behaviors [156]. Furthermore, nearly 50% of orexinergic neurons respond to opioids through the expression of μOR [155], which contributes to the rewarding effects of these drugs (Figure 2).

Many studies have focused on exploring therapeutic potential of targeting the orexin system for the treatment of opioid misuse and abuse, and experimental evidence supports the involvement of the orexin system in opioid addiction. In rodent models, OX_1_R antagonism was able to reduce both the intake and seeking behavior associated with synthetic opioids such as fentanyl and remifentanil [158,159]. In another study by the same group, the use of intermittent access (IntA) to fentanyl self-administration in rats demonstrated that this model induces a robust addiction-like state that is orexin-dependent [161]. IntA to fentanyl led to a greater escalation of fentanyl intake, increased motivation for fentanyl, persistent drug seeking during abstinence, and stronger cue-induced reinstatement compared to rats with short or long access. Importantly, these addiction behaviors were reversed by the administration of the orexin-1 receptor antagonist SB-334867, suggesting that OX_1_R enhances the addictive effects of fentanyl. Furthermore, IntA to fentanyl was associated with a persistent increase in the number of orexin neurons, suggesting that chronic opioid use can lead to an upregulation of orexin-producing cells [161]. Indeed, an increased motivation for fentanyl correlates was found to be correlated with an increase in orexigenic neurons [123]. In the study by Mohammadkhani et al., it was observed that administration of the OX_1_R antagonist SB-334867 reduces addiction to remifentanil, and that its effects can persist beyond the drug’s half-life, particularly when administered directly into the ventral pallidum. This reduction in motivation lasts up to 72 h and also decreases the reinstatement of drug-seeking behavior induced by drug-associated cues [216]. The study by De Sa Nogueira et al. found that in female rats SB-334867 did diminish fentanyl seeking, but not consumption or motivation [217]. There is little literature exploring the role of OX_2_R in addiction to fentanyl. Nonetheless, it has been found that OX_2_R plays an important role in mediating fentanyl effects. A study in which danavorexton (selective OX_2_R antagonist) was administered, a faster emergence from the anesthetic effect of fentanyl was observed [218].

Regarding heroine, it has been found that administration of the OX_1_R selective antagonist SB-334867 reduces heroin self-administration and attenuated reinstatement of extinguished heroin seeking induced by cues, but not by heroin priming, supporting the role of OX_1_R in reward seeking conditioned by cues [219]. In heroin-dependent rats, treatment with the OX_2_R selective antagonist NBI-80713 significantly reduced self-administration, suggesting that OX_2_R may also mediate the negative reinforcement of the drugs that drive compulsive intake [157]. Based on these results, therapeutic strategies comprising dual antagonization of orexigenic receptors (DORAs) could be highly beneficial for combating heroin use disorders. The potent semisynthetic opioid oxycodone has also been investigated, showing differential effects between OX_1_R and OX_2_R blockade. While OX_1_R blockade with SB-334867 was able to reduce oxycodone intake in rats, this effect was not observed after the treatment with OX_2_R selective antagonist TCS-OX2-29 [160].

The differential role of both OX_1_R and OX_2_R in opioid addiction is under debate, although data seem to indicate that the main orexigenic receptor involved in addiction is OX_1_, as several studies prove OX_1_R antagonization to be a sensible approach to reduce seeking and motivation towards opioids. Nonetheless, increasing evidence suggests that OX_2_R may also play a crucial role on opioid and substance use disorders in general, although it seems to be more secondary and there is controversy around its functionality in addiction. Further research into the precise mechanisms of orexin-opioid receptor interactions, including potential heteromerization, could lead to novel therapeutic targets for opioid addiction.

### 6.2. Orexin-Dopaminergic System Interaction

The orexin system, originating from the lateral hypothalamus, exerts a profound influence on the mesolimbic dopamine system, a critical circuit for reward processing and motivation, which is heavily implicated in drug addiction [220,221] (Figure 2). Orexin neurons project extensively to key components of this circuit, including the VTA and the NAc, where they modulate dopaminergic neurotransmission and synaptic plasticity primarily through OX_1_R activation [222], thereby contributing significantly to compulsive drug-seeking behaviors [195,209]. Specifically, orexin can directly activate VTA dopamine neurons and enhance dopamine release in the NAc, a mechanism that strengthens the rewarding properties of drugs and reinforces drug-associated memories [223]. This interaction is not merely transient; orexin has been shown to induce long-lasting changes in synaptic strength within the VTA-NAc pathway, a form of neuroplasticity that underlies the persistent alterations observed in addiction [215]. For instance, orexin signaling can strengthen excitatory synapses onto VTA dopamine neurons, making them more responsive to drug-related cues and promoting the drive to seek drugs [195,224]. This potentiation of dopamine signaling by orexins contributes to the reinforcing effects of drugs of abuse and facilitates drug-seeking behaviors [221]. For instance, studies have shown that orexin can augment responses of VTA dopamine neurons to afferent inputs, especially glutamate, thereby playing a crucial role in the salience attribution to reward-associated cues [198]. The sustained activation of the orexin system, often triggered by stress or drug-associated stimuli, can drive maladaptive neuroplastic changes that perpetuate drug-seeking even in the face of negative consequences, highlighting the orexin-dopamine interaction as a crucial target for understanding and treating addiction [192,209].

Experimental studies have elucidated the direct impact of orexin on VTA dopamine neurons. For example, optogenetic stimulation of LH orexin/dynorphin inputs in the VTA has been shown to potentiate mesolimbic dopamine neurotransmission in the NAc core [223]. This potentiation was accompanied by behavioral changes, including real-time and conditioned place preference, and increased food cue-directed orientation in a Pavlovian conditioning procedure [223]. Importantly, the rewarding effects associated with this optogenetic stimulation were predominantly driven by orexin, as they were blocked by an OX_1_R antagonist but not by a k-opioid receptor (kOR) antagonist, despite the co-release of dynorphin [223]. Understanding the precise mechanisms of orexin-dopamine interactions is vital for developing effective treatments that target the reward circuitry in addiction.

### 6.3. Orexin-Cannabinoid System Interaction

The cannabinoid system, comprising cannabinoid receptors 1 and 2 (CB_1_R and CB_2_R) and their endogenous ligands, also plays a significant role in addiction, particularly in modulating reward, stress, and anxiety [225]. Emerging research highlights a complex interplay between the orexigenic and cannabinoid systems. Both orexin receptors and cannabinoid receptors are GPCRs and have been shown to interact forming heteromeric complexes, which can significantly alter their signaling properties and functional outcomes [226]. This intricate interaction suggests that targeting the orexin-cannabinoid receptor interface could offer novel therapeutic avenues for addiction, especially considering the role of cannabinoids in modulating craving and relapse [226].

Experimental studies have provided direct evidence for the involvement of the orexin system in cannabinoid reward. For instance, a study investigating the effects of orexins on the intravenous self-administration of the synthetic cannabinoid agonist WIN55,212-2 demonstrated that systemic administration of the OX_1_R antagonist SB-334867 reduced WIN55,212-2 self-administration and the maximum effort to obtain an infusion [190]. This role of OX_1_R in the reinforcing and motivational properties of WIN55,212-2 was further confirmed in OX_1_R knockout mice [190]. Additionally, contingent (but not noncontingent) WIN55,212-2 self-administration increased the percentage of orexin cells expressing FosB/ΔFosB in the LH [190]. Furthermore, the enhancement in dopamine extracellular levels in the NAc induced by Δ^9^-tetrahydrocannabinol was blocked in mice lacking OX_1_R, suggesting that orexins modulate cannabinoid reinforcing properties through a dopamine-dependent mechanism [190]. These findings collectively indicate that OX_1_R is a novel target to modulate cannabinoid reward, offering clear therapeutic interest (Figure 2).

The ability of orexins to modulate reward pathways, influence drug-seeking behaviors, and form heteromeric complexes with other GPCRs presents exciting opportunities for the development of novel pharmacotherapies. Further research into the precise molecular mechanisms underlying these interactions will be essential for identifying new therapeutic targets and ultimately improving treatment outcomes for individuals suffering from addiction.

## 7. Orexin Receptor Heteromers as Therapeutic Targets in Addiction

Recent studies have highlighted the phenomenon of receptor heteromerization within the orexin system, particularly the interactions between OX1R and OX_2_R with other receptors, such as CB_1_R and CB_2_R [227,228]. Heteromerization plays a crucial role in modulating the signaling outcomes of individual receptors and can significantly influence their physiological effects. For instance, the formation of receptor heteromers can lead to signaling behaviors that are distinct from those observed when receptors are found in their monomeric form. These effects arise from phenomena such as cross-talk or cross-antagonism, among others, which alter the functionality of the heteromer compared to the activity of the individual receptor. Agonist binding induces cross-conformational changes between receptor protomers and GPCR-associated proteins, including heterotrimeric G proteins and β-arrestins. In this context, new complexes composed of receptors that bind the same ligand but generate opposing signaling effects may emerge as novel pharmacological targets, opening avenues for the development of addiction-focused therapeutic strategies [229].

For example, studies have indicated that the orexin system contributes to cocaine seeking reinstatement through the activation of stress pathways involving the CRF neuropeptide. Central administration of OXA induces a dose-dependent reinstatement of cocaine-seeking behavior, an effect that can be blocked by CRF receptor (CRFR) and OX_1_R antagonists [138]. The formation of CRF_1_R–OX_1_R heteromers has also been described. These heteromers mediate negative cross-talk between OXA and CRF in the VTA, significantly modulating dendritic dopamine release. Moreover, CRF_1_R–OX_1_R heteromers can associate with σ_1_ receptors (σ_1_R) to form CRF_1_R–OX_1_R–σ_1_R complexes, where cocaine binding to σ_1_R induces a long-term disruption of this negative cross-talk, sensitizing VTA neurons to the excitatory effects of both OXA and CRF [230].

As previously mentioned, it has been reported that orexin-1 receptors OX_1_R and CB_1_R can associate to form heterodimers in various types of cells and neuronal tissues [228]. Within these heteromeric complexes, ligands binding to one receptor can influence both the localization and signaling of the other, even in the absence of direct affinity [231]. This type of interaction is particularly relevant in the context of drug abuse. For instance, chronic exposure to the synthetic cannabinoid WIN55,212-2 has been shown to alter the activity of OX_1_R-expressing neurons in the lateral hypothalamus. Moreover, the increase in extracellular dopamine within the NAc, triggered by Δ^9^-tetrahydrocannabinol and closely linked to the reinforcing properties of cannabis, is completely abolished in mice lacking OX_1_R. These findings point in a promising direction: orexin receptor antagonists may represent a valuable pharmacological tool for the treatment of cannabis dependence in humans [198,232].

Another heteromer relevant to addiction involves the interaction of OX_1_R with kOR [233]. While kOR monomers primarily signal via Gi/o pathways [234], their dimerization with OX_1_R may result in “opposite” signaling through Gs pathways [235]. Additionally, it has been proposed that kOR stimulation inhibits OX_1_R activation in dopaminergic neurons [235].

OX_1_R can also form heterodimers with the serotonin 5-HT_1A_ receptor, which give rise to a novel G protein-dependent signaling pathway, without interfering with β-arrestin recruitment to the complex. In the study by Zhang et al., the authors observed that the structural interface of the active 5-HT_1A_R/OX_1_R dimer shifts from TM4/TM5 in the basal state to TM6 in the active conformation. Remarkably, the administration of TM4/TM5 peptides to rats exposed to chronic unpredictable mild stress improved their depression-like emotional state while simultaneously reducing the number of endogenous 5-HT_1A_R/OX_1_R heterodimers in the rat brain [236], and addiction and depression are strongly interconnected.

No direct interaction between dopamine and orexin receptors has been described so far; however, evidence shows that blocking OX_1_R significantly reduces the effects of cocaine on dopaminergic signaling and decreases the motivation to consume cocaine [142]. This finding suggests that a direct interaction between these receptors may exist, highlighting the need for further research on the topic.

Several therapeutic strategies targeting receptor heteromers have been explored. Eluxadoline, a μOR agonist and δ-opioid receptor (δOR) antagonist, has been approved for the treatment of irritable bowel syndrome [237]. In the context of substance use disorders, adenosine receptor 2A (A_2A_R)-D_2_R heteromers have been investigated using D_2_R agonists/A_2A_R antagonists to reduce habit formation associated with chronic psychostimulant use [238]. Additionally, biased ligands have been developed, such as SKF83959, which targets D_1_R-D_2_R heteromers and reduces locomotor sensitization and cocaine-seeking reinstatement, and CYM51010, directed at μOR-δOR heteromers, which provides morphine-comparable analgesia with reduced tolerance and physical dependence [239,240].

These findings underscore the potential of orexin receptor heteromers to exhibit unique signaling properties that are distinct from those of individual receptors. This includes interactions with other GPCRs, which may modulate the therapeutic effects of orexin-targeted drugs [116,211]. Understanding receptor heteromerization could refine the design of compounds that selectively target these complexes, thereby enhancing their specificity and efficacy in addiction treatment.

## 8. Future Directions

The intricate involvement of the orexin system in drug addiction, as elucidated throughout this review, underscores its significant potential as a therapeutic target. While current research has primarily focused on the individual roles of orexin receptors (OX_1_R and OX_2_R) and their antagonists in modulating drug-seeking behaviors and relapse, future investigations should delve deeper into the complex interplay between the orexin system and other neurotransmitter systems, particularly through the lens of GPCR heteromerization. The document highlights the critical interaction between the orexin and dopaminergic systems, where orexin neurons extensively project to key components of the mesolimbic dopamine circuit, such as the VTA and the NAc. This interaction, primarily mediated by OX_1_R activation, modulates dopaminergic neurotransmission and synaptic plasticity, thereby strengthening drug-reward properties and reinforcing drug-associated memories [222,223]. Further research should explore the precise molecular mechanisms underlying these interactions, including the potential formation of heteromers between orexin receptors and dopamine receptors (D_1_, D_2_, D_3_, D_4_ and D_5_), which are also GPCRs. Such heteromers could represent novel signaling units with distinct pharmacological profiles, offering more selective therapeutic avenues than targeting individual receptors. Similarly, the document discusses the orexin system’s interaction with the opioid and cannabinoid systems, both of which are heavily implicated in addiction and involve GPCRs (opioid receptors and cannabinoid receptors, respectively). Given the widespread involvement of the opioid system in pain, reward, and stress, understanding these heteromers could unlock new strategies for managing both addiction and co-occurring conditions.

In conclusion, while the therapeutic potential of orexin receptor antagonists is evident, a deeper understanding of orexin receptor biology is needed, particularly their capacity to form heteromers with other GPCRs involved in addiction (such as dopamine, opioid, and cannabinoid receptors), represents a crucial future direction. Investigating these heteromeric complexes could reveal novel pharmacological targets, leading to the development of more effective and selective treatments for substance use disorders by modulating specific signaling pathways rather than broad receptor blockade. This approach promises to refine our therapeutic strategies, offering hope for more personalized and efficacious interventions in the complex landscape of addiction.

## 9. Conclusions

The implication of the orexin system in various stages of addiction highlights its central role in the neurobiology of substance use disorders. The orexin system appears to act as a key modulator of drug seeking and relapse.

The use of orexin receptor antagonists emerges as a promising strategy for therapeutic intervention. Future studies should focus on receptor-specific functions across various substances and investigate the role of OX_1_R signaling, both independently and in combination with OX_2_R signaling, to gain a deeper understanding of the orexin system in substance use disorders

The capacity of orexins to modulate reward pathways, influence drug-seeking behavior, and interact with other GPCRs through heteromeric complexes opens promising avenues for the development of new pharmacotherapies. Advancing our understanding of the specific molecular mechanisms behind these interactions will be crucial for identifying novel therapeutic targets and ultimately enhancing treatment outcomes for individuals struggling with addiction.

Interactions between orexin receptors and other GPCRs may influence the therapeutic potential of orexin-targeted drugs. A deeper understanding of receptor heteromerization could guide the development of compounds that precisely target these complexes, thus improving both specificity and effectiveness in the treatment of addiction.

Clinical findings in human studies highlight the necessity of targeting the orexin system for the treatment of addiction. Further work unraveling orexin’s network level interactions and addressing safety profiles will be essential for advancing on the therapeutic use of orexin in order to treat addiction.

## Figures and Tables

**Table 1:** Preclinical studies targeting the orexin system in addiction.

Substance of Abuse	Intervention	Subjects	Main Findings	Ref
**Opioids**	Orexin peptide KO	R (rat)	Attenuation of morphine dependence	[155]
	Pre-pro orexin KO	M (mouse)	Abolishment of subcutaneous morphine-induced place preference and hyperlocomotion	[156]
	SB-334867A (selective OX_1_R antagonist)	R	Suppression of morphine-induced place preference	[156]
	NBI-80713 (selective OX_2_R antagonist)	R	Reduction in morphine self-administration	[157]
	SB-334867	R	Decreased motivation for fentanyl	[158]
	SB-334867	R	Decreased motivation for remifentanyl	[159]
	SB-334867	R	Reduction in oxycodone intake	[160]
	SB-334867	R	Reversion of fentanyl-induced addiction state	[161]
	Elimination of orexin neurons	M	Reduction in the somatic and affective symptoms of withdrawal	[162]
	Suvorexant (dual OX_1_/_2_R antagonist)	Humans	Decreased diurnal salivary cortisol levels and self-reported stress in humans undergoing opioid withdrawal	[163]
	Suvorexant	M	Decreased morphine tolerance and dependence/decreased increased levels of CREB and p-ERK proteins	[164]
	SB-334867	M	Prevented morphine-induced sensitivity to locomotor activity in mice	[165]
	SB-334867	R	Significantly reduced naloxone-induced withdrawal syndrome physical symptoms in morphine-dependent rats	[166]
	SB-334867	R	Microinjection into LC dramatically suppresses glutamate-induced morphine withdrawal	[167]
	SB-334867	M	Attenuated the symptoms of naloxone-induced withdrawal	[168]
	SB-334867	R	Attenuation of morphine-induced CPP (acquisition and expression/micro-injection into VTA)	[169]
	SB-334867	R	Intra-DG (dentate gyrus) administration dose-dependently reduced morphine priming-induced reinstatement	[170]
	SB-334867	R	Decreased motivation and the cue-induced reinstatement of remifentanil-seeking	[171]
	SB-334867	R	Inhibition of increased activity of LC neurons following naloxone administration in morphine-dependent rats	[172]
	SB-334867	R	Prevention of naloxone-induced neuronal activation within the LC in morphine-dependent rats/Decreased cAMP concentration in LC neurons	[173]
	SB-334867	R	Significant reduction in physical symptoms of morphine withdrawal syndrome induced by naloxone95	[174]
	TCS-OX2-29 (OX_2_R antagonist)	R	Intra-DG administration dose-dependently reduced morphine priming-induced reinstatement	[170]
	TCS-OX2-29	R	Attenuation of morphine-induced CPP (acquisition and expression/micro-injection into VTA)	[169]
**Alcohol**	SB-334867	R	Reduction in ethanol self-administration and reinstatement	[113]
	Suvorexant	R	Reduced the latency to REM sleep and sleep and slow-wave-sleep (SWS) onset in a dose-dependent manner/produced REM sleep and SWS fragmentation	[175]
	Almorexant (dual OX_1_/_2_R antagonist)	Healthy humans	Almorexant did not affect the pharmacokinetics of ethanol and did not synergize its effects	[176]
	Almorexant	R	Diminished alcohol self-administration (Systemic or VTA administration)	[134]
	Almorexant	R	It did not enhance the sedative effect of alcohol	[177]
	SB-334867	R	Reduced alcohol intake and preference (Intra-NAc infusions)	[178]
	SB-334867	R	Decreased alcohol relapse drinking	[179]
	GSK1059865 (OX_1_R antagonist)	M	Significantly reduced alcohol consumption in ethanol-dependent animals	[180]
	TCS-OX2-29	R	Microinjections of TCS-OX2-29 (into the aPVT) reduced intermittent-access ethanol drinking	[181]
**Cocaine**	SB-334867	R	Blockade of footshock-induced reinstatement of previously extinguished cocaine-seeking behavior	[138]
	SB-334867	R	Reduction in work to self-administer cocaine or high fat food pellets	[182]
	SB-334867	R	Dose-dependent decrease in cue-induced reinstatement of cocaine-seeking	[115]
	SB-334867	R	Blockade of cue-induced reinstatement of cocaine-seeking	[122]
	SB-334867	M	Blockade of CPP induced by micro-injection of orexin in VTA	[183]
	SB-334867	R	Reduced motivation for cocaine	[123]
	OX_1_R knock-down	M	Reduced dopaminergic response to cocaine and motivation to seek the drug	[121]
	Suvorexant	R	Attenuated cocaine-induced impulsive behaviors (systematic or direct injection in VTA)	[143]
	Suvorexant	R	Attenuation of the hedonic and motivational effect induced by cocaine	[184]
	SB-334867	R	Counteracts the development of cocaine self-administration and attenuates the induction of amphetamine-induced CPP	[119]
	SB-334867	R	Decreased cocaine intake (in a dose-dependent manner)	[185]
	SB-334867	M	Attenuated impulsive-like behavior, LH self-stimulation, and cocaine self-administration	[186]
	SB-334867	Female monkeys (rhesus)	Reduced cocaine self-administration	[187]
	SB-334867	R	Blocking OX_1_R or OX_1_R and OX_2_R together reduces the effect of cocaine on dopamine signaling and cocaine motivation, but blocking OX_2_R alone showed no effect	[142]
	Almorexant	R	Decreased cocaine self-administration and weakened cocaine-induced dopamine uptake inhibition	[142]
	RTIOX-276 (OX_1_R antagonist)	R	Attenuation of cocaine-induced inhibition of dopamine uptake	[188]
**Amphetamine**	SB-334867	R	Reduced amphetamine-evoked DA outflow in the NAc and reduced amphetamine-induced sensitization	[118]
	Almorexant	R	Decreased cocaine and amphetamine-induced CPP expression but did not affect morphine-induced CPP expression CPP expression/Interfered with morphine-induced locomotor sensitization but had no effect on cocaine and amphetamine-induced locomotor sensitization	[189]
**Cannabis**	SB-334867	M	Reduced the reinforcing and motivational properties of WIN55,212-2 (TCS-OX2-29 had no effect)	[190]
**Nicotine**	SB-334867	M	Reduced somatic signs of nicotine-induced withdrawal (TCS-OX2-29 had no effect)	[117]
	TCS 1102 (dual OX_1_/_2_R antagonist)	R	No effect on nicotine-seeking behavior	[191]

## Data Availability

No new data were created or analyzed in this study.

## Abbreviations

The following abbreviations are used in this manuscript:

δOR	δ-opioid receptor
κOR	κ-opioid receptor
μOR	μ-opioid receptor
σ_1_R	σ 1 receptor
2-AG	2-arachidonoylglycerol
A_2A_R	Adenosine receptor 2A
AUD	Alcohol use disorder
CB_1_R	Cannabinoid receptor 1
CB_2_R	Cannabinoid receptor 2
CNS	Central Nervous System
CPP	Conditioned place preference
CRF	Corticotropin-releasing factor
CRF_1_R	CRF receptor 1
D_1_R	Dopamine receptor 1
D_2_R	Dopamine receptor 2
DG	Dentate gyrus
DORA	Dual orexin receptor antagonist
GPCRs	G protein-coupled receptors
HPA	Hypothalamic–pituitary–adrenal axis
iCa^2+^	Intracellular calcium
IntA	Intermittent access
KO	Knockout
LC	Locus coeruleus
LDT	Laterodorsal tegmental nuclei
LH	Lateral hypothalamus
LTP	Long-term potentiation
MAPK	Mitogen-activated protein kinase
mTORC1	Mammalian Target of Rapamycin complex 1
NAc	Nucleus accumbens
NMDA	N-methyl-D-aspartate
OXA	Orexin A
OXB	Orexin B
OXRs	Orexin receptors
OX_1_R	Orexin receptor 1
OX_2_R	Orexin receptor 2
PKC	Protein kinase C
PLC	Phospolipase C
PFC	Prefrontal cortex
PPT	Pedunculopontine tegmental nuclei
pPVT	Paraventricular nucleus of the thalamus
TMN	Tuberomammillary nucleus
VGCC	Voltage-gated Ca^2+^ channel
VTA	Ventral tegmental area
